# No detrimental effect of ligament balancing on functional outcome after total knee arthroplasty: a prospective cohort study on 129 mechanically aligned knees with 3 years’ follow-up

**DOI:** 10.1080/17453674.2018.1485283

**Published:** 2018-06-08

**Authors:** Eirik Aunan, Stephan M Röhrl

**Affiliations:** Department of Orthopaedic Surgery, Sykehuset Innlandet Hospital Trust, Lillehammer;; Orthopaedic Department, Oslo University Hospital, Oslo, Norway

## Abstract

**Background and purpose** — In the classical mechanical alignment technique, ligament balancing is considered a prerequisite for good function and endurance in total knee arthroplasty (TKA). However, it has been argued that ligament balancing may have a negative effect on knee function, and some authors advocate anatomic or kinematic alignment in order to reduce the extent of ligament releases. The effect of the trauma induced by ligament balancing on functional outcome is unknown; therefore, the aim of this study was to investigate this effect.

**Patients and methods** — 129 knees (73 women) were investigated. Mean age was 69 years (42–82), and mean BMI was 29 (20–43). Preoperatively 103 knees had a varus deformity, 21 knees had valgus deformity, and 5 knees were neutral. The primary outcome measure was the Knee injury and Osteoarthritis Outcome Score (KOOS). Secondary outcome measures were the Oxford Knee Score (OKS) and patient satisfaction (VAS). All ligament releases were registered intraoperatively and outcome at 3 years’ follow-up in knees with and without ligament balancing was compared

**Results** — 86 knees were ligament balanced and 43 knees were not. Ligament-balanced varus knees had more preoperative deformity than varus knees without ligament balancing (p = 0.01). There were no statistically significant differences in outcomes between ligament-balanced and non-ligament-balanced knees at 3 years’ follow-up. No correlation was found between increasing numbers of soft tissue structures released and outcome.

**Interpretation** — We did not find any negative effect of the trauma induced by ligament balancing on knee function after 3 years.

Symmetric ligament balancing, creating equal and rectangular gaps, has traditionally been considered a prerequisite for good function and endurance in total knee arthroplasty (TKA) (Sharkey et al. 2002, Matsuda et al. 2005, Graichen et al. 2007, Delport et al. 2013). The need for and the extent of ligament balancing is influenced by patient-dependent factors and surgical factors. The most important patient-dependent factors are the degree of knee deformity and the status of the ligaments and other soft tissues around the knee. The predominant surgical factors are the alignment goal, and whether a measured resection technique or a gap technique is used.

3 different principles for alignment exist. Classical mechanical alignment (Insall et al. 1985), anatomic alignment (­Hungerford and Krackow 1985), and kinematic alignment (Hollister et al. 1993, Eckhoff et al. 2005). In mechanical alignment, the aim is to place the center of the femoral and tibial components at the mechanical axis of the lower extremity and the joint line perpendicular to the mechanical axis. In contrast, anatomic and kinematic alignment aim to reestablish the patient’s natural premorbid alignment, that is with the mechanical axis passing on average 8 mm medial to the joint center and the joint line in 2°–3°varus (Paley 2003). Consequently, by using anatomical or kinematic alignment in a varus knee, less angular correction of the bone is needed and the extent of medial ligament releases is reduced. However, the scientific support for anatomical and kinematic alignment is currently scarce and mechanical alignment remains the gold standard (Abdel et al. 2014, Gromov et al. 2014).

The extent of ligament balancing can also be reduced by using a gap technique rather than a measured resection technique. When a measured resection technique is used, ligament balancing is performed both in extension and in flexion. In contrast, with a classical gap technique, ligament balancing is performed only in extension (Insall and Easley 2001).

Hence, the extent of ligament releases in varus knees can be reduced by aiming at anatomical or kinematic alignment and/or by using a gap technique. Nevertheless, a possible downside is that the knee will be left with the mechanical axis passing medially to the center of the knee and the joint line in varus. In return, this will lead to uneven distribution of loads through the medial and lateral compartments of the knee and increased share forces on the interfaces between implants and bone. These factors may possibly threaten the longevity of the prosthetic knee (Ritter et al. 2011, Kim et al. 2014).

The exercise of ligament balancing induces an additional surgical trauma to the knee and it could be hypothesized that this trauma is deleterious to functional outcome after TKA. Each surgeon must choose between mechanical, anatomic, or kinematic alignment techniques and between measured resection and gap technique. The effect of the trauma induced by ligament balancing on functional outcome after TKA has not been described in the literature. However, it is a crucial factor to consider when the surgeon will decide whether to perform ligament balancing or not, and which alignment strategy and gap-balancing strategy to use. Therefore, we investigated the effect of the trauma imposed by ligament balancing on functional outcome after TKA.

## Patients and methods

All patients participating in another prospective, randomized, and double-blind study comparing TKA with and without patellar resurfacing (Aunan et al. 2016) were included in this study. Inclusion criteria were patients less than 85 years old scheduled for TKA because of osteoarthritis. Exclusion criteria were knees with severe deformity defined as: bone deformity to such a degree that the bone cuts would damage the ligamentous attachments on the epicondyles; ligament laxity without a firm end-point or to such a degree that ligament releases on the concave side would result in a need for more than 20 mm polyethylene thickness; the combination of bone deformity and ligament laxity resulting in the need for more than 20 mm polyethylene thickness. Excluded were also knees with posterior cruciate deficiency, inflammatory arthritis, and severe medical disability limiting the ability to walk or to fill out the patient-recorded outcome documents. Also excluded were patients with patellar thickness less than 18 mm measured on calibrated digital radiographs, isolated patello-femoral arthrosis, knees with secondary osteoarthritis (except for meniscal sequelae), and knees with previous surgery on the extensor mechanism. 2 patients died before the 3-year follow-up. In these patients, outcome scores 1 year after the operation were carried forward.

Standard radiographs and standing hip–knee–ankle (HKA) radiographs were taken preoperatively and at follow-up. A knee was considered in neutral alignment when the mechanical axis of the lower extremity passed through the center of the tibial spines of the knee and any deviation was termed varus or valgus deformity according to the definitions recommended by Paley (2003).

### Surgical technique

All knees were operated through a standard midline incision and a medial para-patellar arthrotomy, using a posterior cruciate-retaining prosthesis (NexGen, Zimmer, Warsaw, IN, USA) and measured resection technique. Classical mechanical alignment was aimed for by setting the valgus angle of the femoral component at 5–8 degrees, depending on the hip–knee–femoral shaft angle (HKFS) as measured on preoperative HKA radiographs.

Rotation of the femoral component was decided with the clinical rotational axis (CRA) method, described by Aunan et al. (2017). The tibial component was aligned to the medial third of the tibial tubercle or with a modified self-seeking technique. Ligament balancing was performed using the technique described by Whiteside and colleagues (Whiteside 1999, Whiteside et al. 2000). The aims of the ligament balancing were medial and lateral laxities of 1–3 mm in both extension and 90° of flexion, and equal and rectangular flexion and extension gaps. The indication for ligament balancing was laxities outside these limits. If an important difference in the height of the flexion and extension gap was still observed after ligament balancing, the gaps were corrected according to the contingency table described by Mont et al. (1999). Medial and lateral ligament laxity in extension and 90° of flexion was measured with the spatula method (Aunan et al. 2012, 2015). This method has demonstrated a very high inter-rater reliability with an intraclass correlation coefficient equal to 0.88.

### Outcome measures

The primary outcome measure was the Knee injury and Osteoarthritis Outcome Score (KOOS) (Roos and Toksvig-Larsen 2003). Secondary outcome measures were the Oxford Knee Score (Dawson et al. 1998) and patient satisfaction measured on a visual analog scale (VAS). The primary and secondary outcome measures were recorded preoperatively and at 3 years of follow-up. VAS was recorded at 3 years.

First all ligament releases were registered intraoperatively. Second, outcome scores at 3 years’ follow-up in knees with and without ligament balancing was compared. Third, the change in outcome scores from preoperative to the 3-year follow-up in each group was compared. Fourth, the change in outcome scores for varus knees and valgus knees was analyzed separately. Finally, the correlation between increasing number of ligament releases and functional outcome for all ligament-balanced knees was estimated.

### Statistics

A post hoc sample size calculation was performed with the OpenEpi, Version 3 (http://www.openepi.com/Menu/OE_Menu.htm), open source calculator. The minimal clinically important difference (MCID) in KOOS was set at 10 points and the mean SD of all KOOS sub-scores at 3 years was set at 16. The ratio of sample sizes was set at 0.5, the 2-sided CI at 95%, and the power at 90%. Given these data, the total sample size was calculated to be 122 with 41 in one group and 81 in the other.

Data were checked visually for normality based on histograms. Means or median values are presented depending on the distribution of data. Comparison of mean and median values was performed using the independent-samples t-test for normally distributed data and the Mann–Whitney U-test for skewed variables. Fisher’s exact test was used when analyzing categorical variables. The association between the number of ligaments released and outcome was estimated with Spearman’s correlation analysis. A significance level of 5% was used and the analyses were performed with IBM SPSS 22 software (IBM Corp, Armonk, NY, USA).

### Ethics, funding, and potential conflicts of interest

The patients included in this study was recruited from another randomized and double-blind trial that was approved by the Regional Committee of Research Ethics at the University of Oslo (REK: 1.2007.952) and registered at ClinicalTrials.gov (identifier: NCT00553982). Later additions to the protocol was approved by the same committee (ID number: S-07172d 1.2007.952) and (2010/1678 D 33-07172b 1.2007.952 with changes 05.03.2012). All the patients signed an informed consent form. The first author has received funding from Sykehuset Innlandet Hospital trust. There are no conflicts of interest.

## Results

129 knees were investigated (Table 1). Preoperatively 103 knees had a varus deformity, 21 knees had valgus deformity, and 5 knees were neutral (Figure). Ligament-balanced varus knees had statistically significantly more preoperative deformity than varus knees without ligament balancing. No other statistically significant differences in baseline data were observed.

**Figure:**
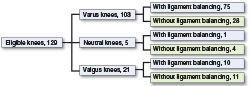
Number of knees with and without ligament balancing in different alignment groups.

**Table 1.** Baseline data for knees with and without ligament balancing. Values are mean (range) unless otherwise specified

Factor	With ligament balancing	Without ligament balancing	p-value
				(n = 86)	(n = 43)
All knees:			
Age	69 (42–81)	70 (53–82)	0.4 ^a^
BMI	29 (23–43)	29 (20–38)	0.8 ^a^
Women/men, n	50/36	23/20	0.7 ^b^
Patellar resurfacing			
yes/no, n	40/46	23/20	0.5 ^b^
Varus knees:			
Number of knees	75	28	
Age	70 (48–81)	70 (53–82)	0.9 ^a^
BMI	29 (23–43)	30 (22–38)	0.4 ^a^
Women/men, n	41/34	13/15	0.5 ^b^
Deformity ^c^	10° (4.4) 2–22	7° (5.1) 1–21	0.01 ^a^
Patellar resurfacing			
yes/no, n	38/37	16/12	0.7 b
Valgus knees:			
Number of knees	10	11	
Age	65 (42–79)	72 (63–82)	0.1 ^a^
BMI	32 (26–38)	28 (20–34)	0.06 ^a^
Women/men, n	9/1	8/3	0.6 ^b^
Deformity ^c^	5° (3.2) 2–13	7° (3.0) 3–13	0.3 ^a^
Patellar resurfacing			
yes/no, n	2/8	6/5	0.2 ^b^
Neutral knees:			
Number of knees	1	4	
Age	69	70 (65–79)	
BMI	32	30 (25–34)	
Women/men, n	0/1	2/2	
Patellar resurfacing			
yes/no, n	0/1	1/3	

^a^Independent samples t-test.

^b^Fisher’s exact test.

^c^Mean (SD) and range

86 knees were ligament balanced and 43 knees were not. In the ligament-balanced knees, mean 2 (1–4) ligament structures were released per knee (Table 2).

**Table 2.** Frequency of soft tissue releases in 86 ligament-balanced knees

Structure released	Varus knees	Valgus knees	Neutral knees
MCL, anterior part	57	2 ^a^	1
MCL, posterior part	47	1 ^a^	0
Medial posterior capsule	11	0	0
Semimembranosus	2	0	0
Pes anserinius	0	0	0
Popliteus tendon	5	4	0
Lateral collateral ligament	1	1	0
Tractus ileotibialis	0	4	0
Posterior-lateral corner.	0	2	0
Lateral posterior capsule	0	4	0
Posterior cruciate ligament	33	3	0
Total	156	21	1

^a^Compensatory releases.

MCL: Medial collateral ligament.

There were no statistically significant differences in outcome scores between ligament-balanced and non-ligament-balanced knees at 3 years’ follow-up (Table 3), or in change of outcome score from baseline to follow-up between the 2 groups (Table 4). When varus and valgus knees were investigated separately, still no difference between ligament-balanced and non-ligament-balanced knees was observed (Table 5). No correlation was found between increasing numbers of soft tissue structures released on the one hand and KOOS, OKS or patient satisfaction on the other.

**Table 3.** Median (IQR) values for functional outcome for ligament-balanced and non-ligament-balanced knees at 3 years follow-up

Factor	Without ligament balancing	With ligament balancing	p-value ^a^
				(n = 43)	(n = 86)
KOOS:			
Pain	92 (17)	97 (19)	0.3
Symptoms	89 (14)	93 (14)	0.9
ADL	93 (24)	94 (24)	0.7
Sport/recreation	70 (45)	65 (41)	0.9
QOL	88 (38)	88 (27)	0.9
Oxford Knee Score	56 (10)	57 (7)	0.3
Patient satisfaction	98 (10)	98 (10)	0.6

^a^Mann-Whitney U test.

KOOS: Knee injury and Osteoarthritis Outcome Score, 0–100.

Best score is 100. ADL: Activities of daily living. QOL: Knee related quality of life.

Oxford knee score, 12-60. Best score is 60.

**Table 4.** Mean (SD) change in outcome scores for all knees (N = 129) from baseline to the 3 years follow up in ligament-balanced and non-ligament-balanced knees

Factor	Without ligament balancing	With ligament balancing	p-value ^a^
	(n = 43)	(n = 86)	
KOOS:			
Pain	42 (18)	48 (19)	0.09
Symptoms	36 (17)	37 (20)	0.7
ADL	38 (19)	42 (21)	0.3
Sport/recreation	48 (27)	49 (30)	0.8
QOL	55 (22)	58 (25)	0.5
Oxford Knee Score	18 (7)	20 (8)	0.4

^a^Independent samples t-test.

Abbreviations: See Table 3.

**Table 5.** Mean (SD) change in outcome scores from baseline to the 3 years follow up for varus-deformed and valgus-deformed knees in ligament-balanced and non-ligament-balanced knees

Factor	Without ligament balancing	With ligament balancing	p-value ^a^
	(n = 43)	(n = 86)	
**Varus knees (n = 103), n**	**28**	**75**	
KOOS:			
Pain	46 (19)	49 (18)	0.6
Symptoms	37 (16)	36 (20)	0.9
ADL	40 (21)	41 (20)	0.8
Sport/recreation	52 (26)	50 (29)	0.7
QOL	60 (20)	58 (25)	0.7
Oxford Knee Score	20 (8)	20 (8)	1.0
**Valgus knees (n = 21), n**	**11**	**10**	
KOOS:			
Pain	35 (12)	45 (26.)	0.3
Symptoms	38 (12)	41 (18)	0.7
ADL	37 (11)	45 (22)	0.3
Sport/recreation	44 (25)	42 (33)	0.8
QOL	49 (20)	56 (31)	0.6
Oxford Knee Score	15 (5)	19 (11)	0.3

^a^Independent samples t-test.

Abbreviations: See Table 3.

## Discussion

Our findings indicate that the surgical trauma imposed by ligament balancing does not have a detrimental effect on knee function as assessed 3 years after the operation. The majority of the ligament-balanced knees had more deformity at baseline than the non-ligament-balanced knees, indicating a less favorable prognosis. Nevertheless, despite multiple releases in many knees, we could not find any negative effect of ligament balancing.

It is well documented that as much as one-fifth of TKA patients are unsatisfied with their TKA (Bourne et al. 2010). The majority of TKAs have until now been aligned according to the principle of mechanical alignment. However, it has been shown that most native knees are slightly varus-aligned (Paley 2003) and that one-third of men and one-fifth of women have constitutional varus knees with a natural mechanical alignment ≥3° degrees varus (Bellemans et al. 2012). Based on this information, it has been speculated that one reason for dissatisfaction with TKA can be that mechanical alignment does not recreate the patient’s premorbid natural alignment (Bellemans et al. 2012, Lee et al. 2017), and that the increased need for ligament balancing in mechanically aligned varus knees can be detrimental to the functional outcome (Bellemans et al. 2012, Gu et al. 2014). Our findings do not support this theory, indicating that the need for additional soft tissue releases is not a valid argument against mechanical alignment in TKA.

Kinematic alignment reduces the need for ligament and other soft tissue releases in 2 different ways: first, in traditional mechanical ligament balancing the goal is to obtain rectangular and equal flexion and extension gaps (Sharkey et al. 2002, Matsuda et al. 2005, Graichen et al. 2007, Delport et al. 2013). In kinematic alignment theory, the aim is to restore the native laxity of the knee ligaments (Lee et al. 2017). Native knee ligament laxity is more pronounced laterally than medially and more laxity is present in flexion than in extension (Tokuhara et al. 2004, Van Damme et al. 2005, Nowakowski et al. 2012). Consequently, by preserving these native properties the need for medial soft tissue releases in a varus-deformed knee is reduced as compared with traditional mechanical balancing. Second, in kinematically and anatomically aligned TKAs the need for soft tissue releases in varus deformed knees is reduced because less correction of the varus deformity is needed, thus less tension is generated in the medial soft tissues.

The degree of ligament balancing in flexion can also be reduced if a gap technique is used instead of a measured resection technique (Insall and Easley 2001). However, in a varus knee this will lead to external rotation of the femoral component and varus alignment in flexion. In a valgus knee, it will result in internal rotation of the femoral component and potential maltracking of the patella and valgus deformity in flexion.

Mechanical alignment is still considered a gold standard (Abdel et al. 2014, Gromov et al. 2014) but anatomic and kinematic alignment have gained increasing popularity in the last decade (Lee et al. 2017) and there is an ongoing debate as to what is the best alignment goal (Lee et al. 2017, Young et al. 2017). Classical mechanical alignment was introduced in order to secure equal distribution of loads between the medial and lateral compartments of the knee and to reduce shear forces at the interfaces between implants and bone (Insall et al. 1985). However, some recent studies have failed to show a relationship between coronal plane alignment and prosthetic survival (Parratte et al. 2010, Bonner et al. 2011). Therefore, in the hope of improving knee function after TKA growing enthusiasm for anatomic and kinematic alignment has emerged. Nevertheless, an important matter to consider is the ability of current surgical techniques to reach the exact alignment goal. Although outliers from the mechanical axis ≥3° may be acceptable (Parratte et al. 2010, Bonner et al. 2011), the same amount of divergence in varus from the natural axis is probably not compatible with long-term survival and good knee function. Consequently, in order to prevent unacceptable outliers, the use of anatomic or kinematic alignment presumes surgical techniques with a high degree of accuracy and precision. Another limitation to the kinematic alignment theory is that replication of normal alignment and ligament laxity does not necessarily lead to more natural knee joint kinematics in TKA. It must be remembered that almost all total knee designs sacrifice 1 or both cruciate ligaments. The lack of well-functioning cruciate ligaments has profound impact on knee kinematics (Scanian and Andriacchi 2017), and non-anatomic prosthetic design features are needed to compensate for the lack of the cruciate ligament(s) and secure stability. It is therefore the authors’ opinion that, in the current context, the term kinematic alignment is too optimistic.

There are some limitations to this study. First, when the study population was subdivided into varus- and valgus-deformed knees (Table 5) the subsequent comparisons between ligament balanced and non-ligament balanced knees are underpowered, increasing the risk of a type 2 error. However, we observed no trends in favor of the non-ligament-balanced knees. Second, we do not know how the ligament-balanced knees would have performed without ligament balancing. Nevertheless, the fact that no differences between the groups were found in change in scores (Δ-values) (Tables 4 and 5) and that no correlation was found was found between increasing numbers of released soft tissue structures and outcome suggests that no real difference between the groups exists. Although an RCT could have been preferred, given the huge amount of literature pointing out the importance of proper ligament balancing in deformed knees with soft tissue contractures, it is our opinion that an RCT on this population would be unethical. Third, ligament balancing was performed according to the methods described by Whiteside et al. (Whiteside 1999, Whiteside et al. 2000). The results of our study are therefore not valid for other ligament-balancing techniques. Finally, optimal ligament balancing has until recently been unknown. Some earlier reports that compared lax and tight TKAs found better functional outcomes in lax knees (Edwards et al. 1988, Kuster et al. 2004). However, during the last decade different research groups have come to conclusions or recommendations that seem to resemble each other. For example, Heesterbeek et al. (2008) recommended 0.7–3.9° valgus laxity and 0.2–5.4° varus laxity in extension. In flexion they recommended between 0° and 7.1° varus laxity and between 0° and 5.5° valgus laxity. Bellemans et al. (2010) assumed ligament balance successful when 2–4 mm medial–lateral joint line opening was obtained in extension and 2–6 mm in flexion. Okamoto et al. (2014) concluded that the extension gap needs more than 1 mm laxity to avoid postoperative flexion contracture in a clinical study. Our research group studied the effect of ligament laxity measured intraoperatively on functional outcome at 1-year follow-up (Aunan et al. 2015). We concluded that medial laxity more than 2 mm in extension and 3 mm in flexion should be avoided in neutral and valgus-aligned knees and that the lateral side is more forgiving. These findings are supported by a recent study by Ismailidis et al. (2017) that found a positive effect on postoperative flexion and patient satisfaction in knees where the flexion gap exceeded the extension gap by 2.5 mm. Furthermore, Tsukiyama et al. (2017) reported that medial rather than lateral knee instability correlates with inferior patient satisfaction and knee function after TKA.

In summary, the potential detrimental effect of the surgical trauma imposed by ligament balancing is an important determinant that must be considered when surgeons choose between different principles for alignment and gap balancing. It is also a crucial factor in cases where the need for ligament releases is debatable. We did not find any negative effect of ligament balancing on knee function after 3 years.

EA: conception, design, data collection, analysis, interpretation, and writing of manuscript. SMR: Revision and approval of the manuscript.

*Acta* thanks Kirill Gromov and Kjell G Nilsson for help with peer review of this study.
